# Fertility preservation after gonadotoxic treatments for cancer and autoimmune diseases

**DOI:** 10.1186/s13048-023-01250-x

**Published:** 2023-08-10

**Authors:** Saki Saito, Mitsutoshi Yamada, Rika Yano, Kazuko Takahashi, Akiko Ebara, Hiroe Sakanaka, Miho Matsumoto, Tomoko Ishimaru, Hiroki Utsuno, Yuichi Matsuzawa, Reina Ooka, Mio Fukuoka, Kazuhiro Akashi, Shintaro Kamijo, Toshio Hamatani, Mamoru Tanaka

**Affiliations:** 1 Department of Obstetrics and Gynecology, Keio University School of Medicine, 35 Shinanomachi, Shinjuku-Ku, Tokyo, 160-8582 Japan; 2 Department of Nursing, Keio University Hospital, 35 Shinanomachi, Shinjuku-Ku, Tokyo, 160-8582 Japan; 3 Clinical Laboratory, Keio University Hospital, 35 Shinanomachi, Shinjuku-Ku, Tokyo, 160-8582 Japan

**Keywords:** Cancer, Autoimmune disease, Fertility preservation, Gonadotropin-releasing hormone analogue, Progestin-primed ovarian stimulation, Female infertility, Cryopreservation, Oocyte quality, Embryo quality, Remission

## Abstract

**Background:**

The indications for fertility preservation (FP) have expanded. A few patients who underwent gonadotoxic treatment did not have the opportunity to receive FP, leading to concerns that these patients may develop premature ovarian insufficiency. However, the usefulness of FP in women with reduced ovarian reserve has also been questioned. Progestin-primed ovarian stimulation can improve the controlled ovarian stimulation (COS) protocol, but there is limited data on the efficacy of FP with progestin-primed ovarian stimulation.

**Methods:**

We conducted a prospective study of 43 women with cancer or autoimmune diseases before and after gonadotoxic treatment at the reproductive unit of Keio University Hospital, counselled between 1 January 2018 and 31 December 2021. After counselling, informed consent was obtained for FP from 43 patients, with those who underwent gonadotoxic treatment of the primary disease being prioritised. Gonadotropin-releasing hormone analogue or progestin was used to suppress luteinising hormone in COS before or after gonadotoxic treatment. The number of cryopreserved mature oocytes was the primary outcome.

**Results:**

Forty-three patients and 67 assisted reproductive technology cycles were included in the analysis. The median age at entry was 32 [inter quartile range (IQR), 29–37] years. All patients in the post-gonadotoxic treatment group had their oocytes frozen. Gonadotoxic treatment resulted in fewer oocytes [median 3 (IQR 1–4); pre-gonadotoxic treatment group: five patients, 13 cycles] vs. median 9 (IQR 5–14; pre-gonadotoxic treatment group: 38 patients, 54 cycles; *P* < 0.001). Although anti-Müllerian hormone levels were lower in the post-gonadotoxic treatment group (*n* = 5, 13 cycles, median 0.29 (IQR 0.15–1.04) pg/mL) than in the pre-gonadotoxic treatment group (*n* = 38, 54 cycles, median 1.89 (IQR 1.15–4.08) pg/mL) (*P* = 0.004), oocyte maturation rates were higher in the post-gonadotoxic treatment group [median 100 (IQR 77.5–100) %] than in the pre-gonadotoxic group [median 90.3 (IQR 75.0–100) %; *P* = 0.039]. Five patients in the pre-gonadotoxic treatment group had their cryopreserved embryos thawed, of which three had live births.

**Conclusions:**

Oocytes obtained for FP from women with cancer or autoimmune disease for FP are of satisfactory quality, regardless of whether they are obtained post-gonadotoxic treatment or COS protocols.

**Supplementary Information:**

The online version contains supplementary material available at 10.1186/s13048-023-01250-x.

## Background

Improving survivorship in adolescents and young adults with cancer is challenging. Since the first guideline for fertility preservation (FP) was published in 2006 [1] and revised in 2018 [2], the indications for FP therapy have expanded. As evidence on the efficacy and safety of oocyte freezing for FP has increased, the freezing of oocytes from patients with cancer is no longer deemed experimental by the American Society for Reproductive Medicine as of October 2012. The latest guidelines of both the American Society of Clinical Oncology and American Society for Reproductive Medicine state that “health care providers should initiate the discussion on the possibility of infertility with patients with cancer treated during their reproductive years or with parents/guardians of children as early as possible” [2].

The therapy is becoming common, as demonstrated in a survey of its status [3] after guidelines for FP in patients with cancer were published in 2017 in Japan [4–6]. According to the survey, 2,537 oocytes or embryos and 178 ovarian tissues were cryopreserved between 2016 and 2019 (1,085 and 122, respectively, between 2011 and 2015). In a survey of 191 members of the Oncofertility Consortium Global Partners Network, funded by the National Institutes of Health, 37 of 40 respondents (93%) identified barriers to care, with a lack of insurance coverage and significant financial burden on patients as the most frequent factors (both 62%, 23 of 37) [7]. In Japan, the public subsidy system started by the Ministry of Health, Labour and Welfare in 2021, which compensates up to 200,000 yen for each unfertilised oocyte frozen and 350,000 yen for each of two fertilised oocytes from patients under the age of 43 years, can enable the post-gonadotoxic treatment group to undergo FP.

Gonadotoxic treatment can be provided to patients with cancer and autoimmune diseases, making FP challenging. A few patients who underwent gonadotoxic treatment, before the guidelines published in 2006 [1] were widely available, did not have the opportunity to receive FP, leading to concerns that these patients may develop premature ovarian insufficiency (POI). A Scottish study involving large groups of patients reported lower pregnancy and first pregnancy rates in cancer survivors compared with patients with non-cancer diseases (standard incidence ratio 0.62, adjusted hazard ratio 0.57) [8]. In addition, the average age of first childbirth in developed countries has been increasing for decades; for example, in 2020, the average age of first childbirth in Japan was 30.7 years [9]. Patients subjected to gonadotoxic treatment tend to give birth later in life; therefore, it is suggested that FP is needed after gonadotropin treatment because of the possibility of the additional effect of declining reproductive function with age. However, the European Society of Human Reproduction and Embryology guidelines question the usefulness of FP in women with reduced ovarian reserve (serum anti-Müllerian hormone (AMH) < 0.5 ng/mL) [10].

The FP therapy needs to be performed within 2–3 weeks after diagnosis to avoid delays in treatment for the underlying disease. Random start gonadotropin-releasing hormone antagonist (GnRH-ant) protocols are widely used in FP because the time to oocyte retrieval is extremely short, allowing for oocyte retrieval within a short period [11, 12]. Progestin-primed ovarian stimulation (PPOS) is helpful for controlled ovarian stimulation (COS) and for suppressing unexpected increases in luteinising hormone (LH) [13]. PPOS is gaining increasing attention because of its simplicity and low cost. However, evidence on the efficacy and safety of the random start PPOS protocols is lacking [14].

In this study, we examined the efficacy of FP involving cryopreservation of oocytes and embryos obtained using different COS protocols in patients with malignancies or autoimmune diseases before and after gonadotoxic treatment.

## Materials and methods

### Population

We conducted a prospective cohort study to evaluate data on FP treatment cycles among patients with cancer or autoimmune disease before and after gonadotoxic treatment at the reproductive unit of Keio University Hospital in the period from January 2018 to December 2021.

**Fig. 1:**
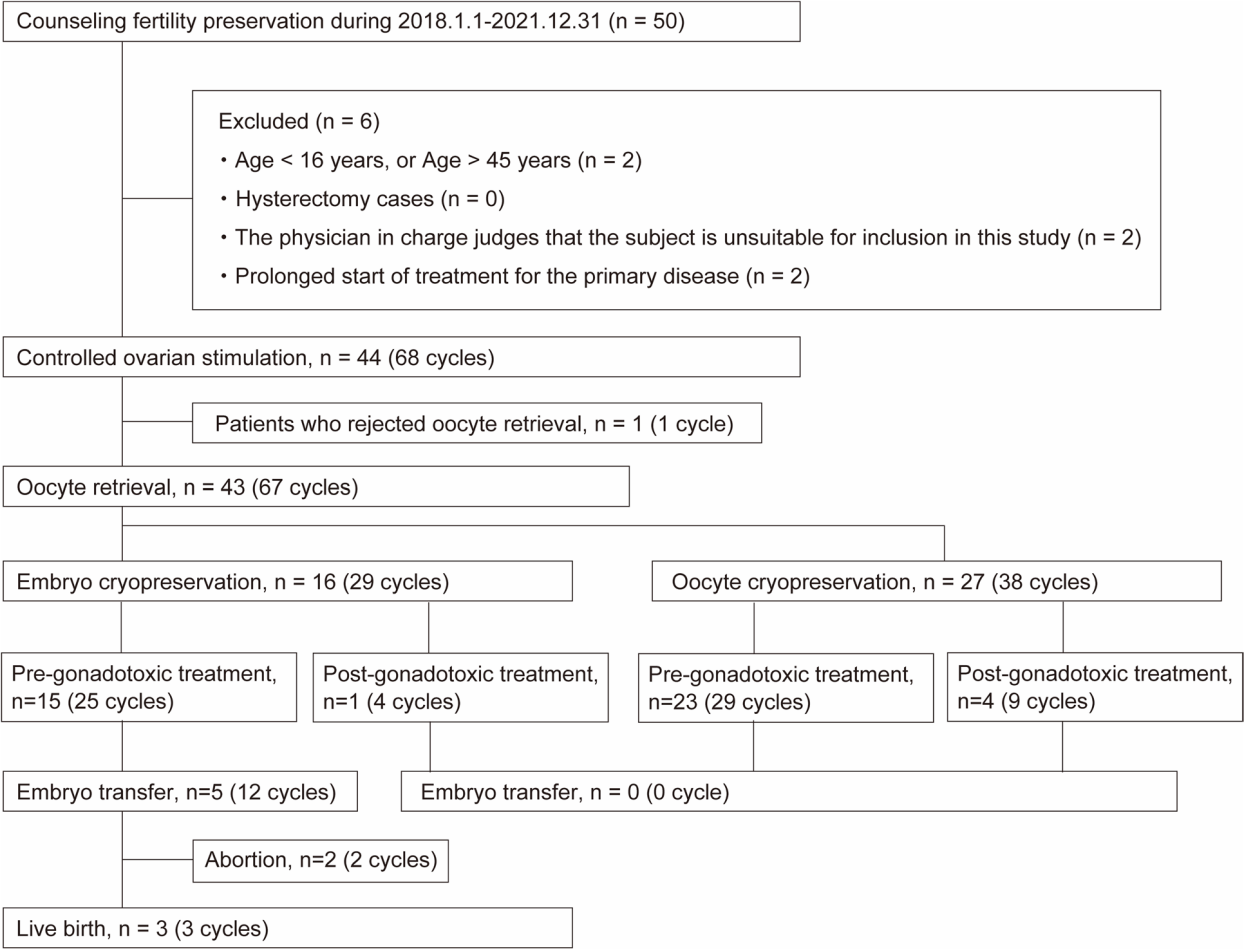
Flowchart of patient selection process. Women with cancer or autoimmune disease consulted on fertility preservation (FP) options at Keio University Hospital during January 2018 and December 2021 were enrolled

The exclusion criteria were as follows: 1) age: < 16 years or > 45 years; 2) patients who had undergone a hysterectomy; 3) patients who did not provide consent; 4) patients judged as inappropriate for inclusion in this study by the physician in charge, and 5) patients for whom the start of treatment for the primary disease would be delayed (Fig. 1).

### Study groups

Patients who underwent FP before gonadotoxic treatment for cancer or autoimmune disease were defined as the pre-gonadotoxic treatment group, and patients who underwent FP after gonadotoxic treatment were defined as the post-gonadotoxic treatment group. Data on all patients were collected from medical records, including data regarding age, antral follicle count (AFC), baseline level of follicle-stimulating hormone, AMH, type of cancer and autoimmune disease, stimulation protocol, gonadotropin dose, type of trigger, number of oocytes retrieved and cryopreserved, rate of fertilisation, and number of frozen embryos. Reproductive toxicity from chemotherapy and radiation therapy was determined based on the risk classification described by the American Society of Clinical Oncology Guidelines 2013 [2].

### Controlled ovarian stimulation protocol

We performed early follicular phase stimulation (days 2–3) in 31 cycles, luteal phase stimulation (random-start) in 31 cycles, and double stimulation in the same COS cycle (DuoStim) [15] in five cycles. The following COS protocols were performed, i.e., 27 cycles of the GnRH-ant protocol; 17 cycles of PPOS; and 23 cycles of other protocols, consisting of 6, 1, 1, and 15 cycles of the short gonadotropin-releasing hormone analogue (GnRHa) protocol, long GnRHa protocol, clomiphene citrate (Clomid; Fuji Pharma, Toyama, Japan) only, and combined protocols, respectively. In the GnRH-ant protocol, 0.25 mg GnRH-ant (Cetrotide, MerckBiophama, Darmstadt, Germany) was administered daily, starting on the day when the dominant follicle diameter reached 14 mm. In the PPOS group, 20 or 30 mg of dydrogesterone (Duphaston, Mylan, Tokyo, Japan) was administered each day from the start of COS until ovarian triggering. In the short GnRHa protocol, GnRHa was administered on the first day of gonadotropin stimulation, i.e., on the first or second day of menstruation, until ovarian triggering. However, in the long GnRHa protocol, GnRHa was administered from the mid-luteal phase or the second day of the previous menstrual cycle until ovarian triggering. The COS protocol involving gonadotropins was initiated following GnRHa administration for more than 2 weeks or when serum estradiol was below 30 pg/mL after the start of menstruation during the oocyte retrieval cycle.

Final oocyte maturation was triggered by subcutaneous injection of 0.25 mg recombinant human chorionic gonadotropin (Ovidrel; EMD Serono, Rockland, MA, USA) or nasal spray of 1 mg of GnRHa buserelin (Suprefact; Sanofi-Aventis, Paris, France). Ultrasound-guided oocyte retrieval was performed 34–36 h later under conscious sedation using an 18–21-gauge single-lumen needle (Kitazato OPU needle; Kitazato Corp., Shizuoka, Japan).

The aspirated oocytes were cryopreserved and included either unfertilised oocytes or oocytes fertilised by conventional insemination or intracytoplasmic sperm injection. All oocytes and embryos were cryopreserved using the Cryotop carrier system (Kitazato Corp.), using a vitrification technique [16, 17].

### Outcomes and definitions

The primary outcome of the current study was the number of retrieved mature oocytes [metaphase II (MII) oocytes]. The secondary outcomes included the total number of oocytes retrieved, rate of oocyte maturation (number of MII oocytes/ retrieved oocytes), number of vitrified MII oocytes, rate of fertilisation (number of fertilised oocytes/MII oocytes), rate of cleavage development (number of day-3 embryos/ fertilised oocytes), rate of blastocyst development (number of blastocysts/ fertilised oocytes), and number of vitrified embryos.

Degenerated oocytes were excluded from the count of oocytes collected when the zona pellucidae was found to be damaged at the time of oocyte inspection with evident cytoplasmic degeneration. However, oocytes that degenerated post-denuding or post-fertilisation were included in the count.

All oocytes were frozen regardless of their maturity level, i.e., fertilised oocytes that reached the blastocyst stage after day-5. However, a few fertilised oocytes that showed partial degeneration or blastocysts assigned a C grade based on the Gardner classification were discarded without freezing after consultation with the embryologist and physician, considering the patient background and embryo details.

### Statistical analyses

All data were analysed using SPSS Statistics for Mac (version 26.0; SPSS, Inc., Chicago, IL, USA), Excel software (Microsoft Corp., Redmond, WA, USA), R software (version 4.2.1; The R Project for Statistical Computing, Vienna, Austria), and GraphPad Prism software (GraphPad, Inc., San Diego, CA, USA). *P*-value < 0.05 was considered to indicate statistical significance. Data are presented as the median (with interquartile range) for normally distributed or skewed data. Student’s *t*-test was used for normally distributed data, and Wilcoxon’s test was used for skewed data. The correlation between age, AMH, and assisted reproductive technology (ART) outcome was analysed using linear regression analysis. As several patients’ characteristics statistically differed between the pre- and post-gonadotoxic treatment groups, a multivariate general linear model (GLM) was used to adjust for the confounding factors.

## Results

### Patient characteristics and ART outcome after FP

Fifty-six patients with cancer or autoimmune diseases were enrolled in this study. Among these, 44 patients met the inclusion criteria, and 43 patients were included for analysis (one patient did not undergo oocyte retrieval after initiation of COS for personal reasons). A total of 67 COS cycles were performed, which comprised 29 in vitro fertilisation cycles in 16 patients and 38 oocyte freezing cycles in 27 patients between January 2018 and December 2021.

Five patients in the pre-gonadotoxic treatment group underwent nine thawed embryo transfers in total (eleven blastocysts) after embryo freezing; all five patients achieved clinical pregnancy, and three of the five patients had live births, resulting in a clinical pregnancy rate of 55.6% (5/9) per embryo transfer. Among the five patients who demonstrated clinical pregnancy, two had miscarriages (40.0%, 2/5), one had a preterm delivery (20.0%, 1/5), and two had full-term deliveries (40.0%, 2/5). Each patient underwent 1 (1–2) oocyte retrieval cycle, with 11 (9–17) mature oocytes retrieved from each patient. Of these, 9 (3–12) oocytes were fertilised, 4 (3–7) of these were frozen, and 2 (2–2) of these were used for transfer. None of the patients who chose to freeze unfertilised oocytes thawed the oocytes during the study period (Table 1).

**Table 1:** Participants' characteristics and ART outcome

**Characteristics**		
Number of participants (n)	43	
Number of cycles (n)	67	
Age (years)	32	(29–37)
AFC	7	(4–13)
Basal FSH (IU/L)	6.1	(5.7–9.9)
AMH (ng/ml)	1.48	(0.29–3.22)
Disease type [n (%)]^a^		
Haematological cancers	6	(14.0)
Breast cancer	22	(51.2)
Gynecological cancers	3	(7.0)
Sarcoma	2	(4.7)
Other cancers	6	(14.0)
Systemic autoimmune diseases	4	(9.3)
Gonadotroxic treatment [n (%)]^a^		
Before fertility preservation	38	(88.4)
After fertility preservation	5	(11.6)
Stimulation start timing/ stimulation phase [n (%)]^b^		
Normal (Day 1–4)	31	(46.3)
Random (Follicular phase, Luteal phase)	31	(46.3)
DuoStim	5	(7.5)
LH suppression regimen [n (%)]^b^		
GnRH antagonist	27	(40.3)
PPOS	17	(25.4)
Others	23	(34.3)
Gonadotropin		
Starting dose of Gonadotropin (IU)	225	(150–300)
Duration of Gonadotropin use (days)	10	(8–12)
Total dosage of Gonadotropin use (IU)	2100	(1350–2700)
Triggar [n (%)]^b^		
hCG or recombinant hCG	64	(95.5)
GnRHa	1	(1.5)
Dual triggar	2	(3.0)
Serum estradiol on trigger day (pmol/L)	1281.5	(749.3–2044.3)
Outcome of oocyte retrieval		
Number of oocyte retrieved (n)	7	(3–12)
Number of MII oocytes retrieved (n)	6	(2–11)
Number of MI oocytes retrieved (n)	0	(0–1)
Number of GV oocytes retrieved (n)	0	(0–1)
Mature oocyte rate (%)^c^	91.7	(76.3–100)
Method for fertilisation [n (%)]^b^		
Conventional insemination	6	(9.0)
Intracytoplasmic sperm injection	14	(20.9)
Split	6	(9.0)
None	41	(61.2)
Embryological outcome		
Fertilisation rate (%)^d^	75.0	(57.1–100)
Cleavage developmental rate (%)^e^	85.7	(62.5–100)
Blastocyst developmental rate (%)^f^	87.5	(57.1–100)
Vitrified stage [n (%)]^a^		
Oocyte	27	(62.8)
Blastocyst	16	(37.2)

Data are median (quartile) unless stated otherwise

*AFC* Antral follicle count, *AMH* Anti-Mullerian hormone, *FSH* Follicle stimulating hormone, *LH* Luteinizing hormone, *GnRH* Gonadotrophin-releasing hormone, *PPOS* Progestin-Primed Ovarian Stimulation, *hCG* Human chorionic gonadotropin, *GnRHa* Gonadotrophin-releasing hormone analogue, *MI/II* Metaphase I/II, *GV* Germinal vesicle

^a^ / participants, ^b^ / cycles, ^c^ MII oocytes / retrived oocytes, ^d^ fertilised oocytes / MII oocytes, ^e^ 8-cells (> day3 6-cells) / fertilised oocytes, ^f^ Blastocysts / fertilised oocytes

### Outcomes of ART before and after gonadotoxic treatment

Patient characteristics and ART outcomes pre- and post-gonadotoxic treatment were compared to examine the impact of gonadotoxic treatments on fertility (Table 2). There were 38 patients (54 cycles) in the pre-gonadotoxic treatment group and five patients (13 cycles) in the post-gonadotoxic treatment group. Patients’ age [33 years (29–37) vs. 26 years (23–40), *P* = 0.026], ovarian reserve parameters revealed by AFC [9 (5–15) vs. 3 (2–4), *P* < 0.001], and serum levels of AMH [1.89 (1.15–4.08), 0.29 (0.15–1.04), *P* = 0.004] were significantly higher in the pre-gonadotoxic treatment group than in the post-gonadotoxic treatment group. The proportions of patients subjected to final maturation trigger types and COS protocol were similar between the pre- and post-gonadotoxic treatment groups. The total and starting FSH dosages were significantly higher in the pre-gonadotoxic treatment group than in the post-gonadotoxic treatment group [2,175 (1,519–2,925) IU vs. 1,350 (1,200–1,950) IU, *P* = 0.026; 225 (150–300) IU vs. 150 (150–150), *P* < 0.001]. The number of retrieved oocytes and MII oocytes were significantly higher in the pre-gonadotoxic treatment group than in the post-gonadotoxic treatment group [9 (5–14) vs. 3 (1–4); *P* < 0.001; 8 (3–12) vs. 3 (0–4), *P* = 0.001]. However, the oocyte maturation rates were higher in the post-gonadotoxic treatment group [100 (77.5–100) %] than in the pre-gonadotoxic group [90.3 (75.0–100) %] (*P* = 0.039). All subjects in the post-gonadotoxic treatment group chose to freeze their unfertilised oocytes, and none underwent fertilisation procedures. The background, course, and results of the post-gonadotoxic treatment group are shown in Supplementary Tables 1 and 2.

**Table 2:** Participants' characteristics and ART outcome according to the gonadotoxic treatment

Characteristics	Pre-gonadotoxic treatment		Post-gonadotoxic treatment		*P*-value
Number of participants (n)	38		5		
Number of cycles (n)	54		13		
Age (years)	33	(29–36.75)	26	(23–40)	0.026
AFC	9	(5–15)	3	(2–4)	< 0.001
Basal FSH (IU/L)	6.1	(5.8–9.2)	10.6	(5.0–16.2)	0.004
AMH (ng/ml)	1.89	(1.15–4.08)	0.29	(0.15–1.04)	0.004
Disease type [n (%)]^a^					
Haematological cancers	6	(11.1)	4	(30.8)	
Breast cancer	30	(55.6)	0	(0.0)	
Gynecological cancers	9	(16.7)	0	(0.0)	
Sarcoma	0	(0.0)	4	(30.8)	
Other cancers	7	(13.0)	0	(0.0)	
Systemic autoimmune diseases	2	(3.7)	5	(38.5)	
Stimulation start timing/ stimulation phase [n (%)]^b^					
Normal (Day 1–4)	23	(42.6)	8	(61.5)	
Random (Follicular phase, Luteal phase)	26	(48.1)	5	(38.5)	
DuoStim	5	(9.3)	0	(0.0)	
LH suppression regimen [n (%)]^b^					
GnRH antagonist	18	(33.3)	9	(69.2)	
PPOS	15	(27.8)	2	(15.4)	
Others	21	(38.9)	2	(15.4)	
Gonadotrophin					
Starting dose of Gonadotropin (IU)	225	(150–300)	150	(150–150)	< 0.001
Duration of Gonadotropin use (days)	10	(8–11)	9	(9–16)	0.198
Total dosage of Gonadotropin use (IU)	2175	(1519–2925)	1350	(1200–1950)	0.026
Triggar [n (%)]^b^					
hCG or recombinant hCG	51	(94.4)	13	(100)	
GnRHa	1	(1.9)	0	(0.0)	
Dual triggar	2	(3.7)	0	(0.0)	
Serum estradiol on trigger day (pmol/L)	1334	(795–2099)	835	(438–1035)	0.149
Outcome of oocyte retrieval					
Number of oocyte retrieved (n)	9	(5–14)	3	(1–4)	< 0.001
Number of MII oocytes retrieved (n)	8	(3–12)	3	(0–4)	0.001
Number of MI oocytes retrieved (n)	0	(0–1)	0	(0–0)	0.010
Number of GV oocytes retrieved (n)	0	(0–1)	0	(0–0)	0.018
Mature oocyte rate (%)^c^	90.3	(75.0–100)	100	(77.5–100)	0.039
Method for fertilisation [n (%)]^b^					
Conventional insemination	6	(11.1)	0	(0.0)	
Intracytoplasmic sperm injection	10	(18.5)	4	(30.8)	
Split	6	(11.1)	0	(0.0)	
None	32	(59.3)	9	(69.2)	
Embryological outcome					
Fertilisation rate (%)^d^	76.4	(59.3–100)	N/A		N/A
Cleavage developmental rate (%)^e^	90.0	(59.4–100)	N/A		N/A
Blastocyst developmental rate (%)^f^	87.5	(59.3–100)	N/A		N/A
Vitrified stage [n (%)]^a^					
Oocyte	23	(60.5)	4	(80.0)	
Blastocyst	15	(39.5)	1	(20.0)	

Data are median (quartile) unless stated otherwise

*AFC* Antral follicle count, *AMH* Anti-Mullerian hormone, *FSH* Follicle stimulating hormone, *LH* Luteinizing hormone, *GnRH* Gonadotrophin-releasing hormone, *PPOS* Progestin-Primed Ovarian Stimulation, *hCG* Human chorionic gonadotropin, *GnRHa* Gonadotrophin-releasing hormone analogue, *MI/II* Metaphase I/II, *GV* Germinal vesicle

^a^ / participants, ^b^ / cycles, ^c^ MII oocytes / retrived oocytes, ^d^ fertilised oocytes / MII oocytes, ^e^ 8-cells (> day3 6-cells) / fertilised oocytes, ^f^ Blastocysts / fertilised oocytes

As differences in age, AFC, AMH levels, and total dosage of gonadotropin used between the pre- and post-gonadotoxic treatment groups were statistically significant, a GLM analysis was employed to remove the potential confounding effects of these variables. As AFC correlated closely with the AMH levels (*r* = 0.65), we used only AMH levels in the GLM to avoid multicollinearity. After the GLM analysis, the effects of gonadotoxic treatments on the number of retrieved oocytes and MII oocytes remained statistically significant (*P* < 0.001 and *P* < 0.001, respectively) (Supplementary Tables 3 and 4).

### Fertility preservation using different COS protocols

The clinical and disease characteristics of the study cohorts were analysed to evaluate the effects of the COS protocol on ART outcomes. PPOS is a recently developed and widely used low-invasive protocol for COS; therefore, we compared this method with the GnRH-ant protocol, a standard COS protocol. The post-gonadotoxic treatment group was excluded from the analysis because of its low ovarian reserve, which might have led to bias when comparing the two groups. Comparison of the PPOS group (11 patients, 15 cycles) with the GnRH-ant group (16 patients, 18 cycles) showed that the age [33 (32–35) vs. 34 (30–37), *P* = 0.979] and ovarian reserve parameters (AFC and AMH) [9 (6–15) vs. 10 (5–19), *P* = 0.888; 1.60 (0.99–3.65) vs. 1.96 (1.32–4.77), *P* = 0.287] in COS protocol-based analyses were similar between the two groups (Table 3). The distribution of patients with different malignancy types or autoimmune diseases was comparable between various COS protocol groups.

**Table 3:** Patients' characteristics and ART outcome according to the controlled ovarian stimulation protocol

Characteristics	GnRH antagonist		PPOS		*P*-value
Number of participants (n)	16		11		
Number of cycles (n)	18		15		
Age (years)	34	(30–37)	33	(32–35)	0.979
AFC	10	(5–19)	9	(6–15)	0.888
Basal FSH (IU/L)	6.1	(5.8–9.4)	6.1	(4.2–8.9)	0.663
AMH (ng/ml)	1.96	(1.32–4.77)	1.60	(0.99–3.65)	0.287
Disease type [n (%)]^a^					
Haematological cancers	2	(11.1)	0	(0.0)	
Breast cancer	13	(72.2)	8	(53.3)	
Gynecological cancers	2	(11.1)	2	(13.3)	
Sarcoma	0	(0.0)	0	(0.0)	
Other cancers	0	(0.0)	4	(26.7)	
Systemic autoimmune diseases	1	(5.6)	1	(6.7)	
Stimulation start timing/ stimulation phase [n (%)]^b^					
Normal (Day 1–4)	13	(72.2)	5	(33.3)	
Random (Follicular phase, Luteal phase)	5	(27.8)	8	(53.3)	
DuoStim	0	(0.0)	2	(13.3)	
Gonadotrophin					
Starting dose of Gonadotropin (IU)	150	(150–281)	225	(188–225)	0.498
Duration of Gonadotropin use (days)	10	(8–10)	10	(9–12)	0.475
Total dosage of Gonadotropin use (IU)	1800	(1369–2325)	2175	(1725–2700)	0.319
Triggar [n (%)]^b^					
hCG or recombinant hCG	16	(88.9)	14	(93.3)	
GnRHa	1	(5.6)	0	(0.0)	
Dual triggar	1	(5.6)	1	(6.7)	
Serum estradiol on trigger day (pmol/L)	1035	(762–1464)	681	(525–1404)	0.270
Outcome of oocyte retrieval					
Number of oocyte retrieved (n)	10	(6–15)	8	(5–11)	0.414
Number of MII oocytes retrieved (n)	9	(4–11)	8	(5–10)	0.810
Number of MI oocytes retrieved (n)	1	(0–1)	0	(0–1)	0.196
Number of GV oocytes retrieved (n)	1	(0–1)	0	(0–0)	0.051
Mature oocyte rate (%)^c^	86.7	(71.6–99.0)	100	(91.0–100)	0.052
Method for fertilisation [n (%)]^b^					
Conventional insemination	2	(11.1)	2	(13.3)	
Intracytoplasmic sperm injection	1	(5.6)	4	(26.7)	
Split	4	(22.2)	0	(0.0)	
None	11	(61.1)	9	(60.0)	
Embryological outcome					
Fertilisation rate (%)^d^	60.0	(50.4–68.9)	87.5	(56.3–100)	0.374
Cleavage developmental rate (%)^e^	71.4	(58.3–100)	100	(89.3–100)	0.237
Blastocyst developmental rate (%)^f^	100	(70.8–100)	100	(87.5–100)	0.694
Frozen embryo (n)	4	(3–4)	5	(2–6)	0.768

Data are median (quartile) unless stated otherwise

*GnRH* Gonadotrophin-releasing hormone, *PPOS* Progestin-Primed Ovarian Stimulation, *AFC* Antral follicle count, *AMH* Anti-Mullerian hormone, *FSH* Follicle stimulating hormone, *LH* Luteinizing hormone, *hCG* Human chorionic gonadotropin, *GnRHa* Gonadotrophin-releasing hormone analogue, *MI/II* Metaphase I/II, *GV* Germinal vesicle

^a^ / participants, ^b^ / cycles, ^c^ MII oocytes / retrived oocytes, ^d^ fertilised oocytes / MII oocytes, ^e^ 8-cells (> day3 6-cells) / fertilised oocytes, ^f^ Blastocysts / fertilised oocytes

The proportion of patients who received different final maturation triggers was also similar. The total dose of gonadotropin was comparable between the COS protocols [2175 (1725–-2700) IU vs. 1800 (1369–2325) IU; *P* = 0.319].

Between the PPOS and the GnRH-ant groups, the numbers of retrieved MII oocytes [8 (5–10) vs. 9 (4–11), *P* = 0.810], immature oocytes, both germinal vesicle oocytes [0 (0–0) vs. 1 (0–1), *P* = 0.051] and metaphase I (MI) oocytes [0 (0–1) vs. 1 (0–1), *P* = 0.196], and maturation rate [100 (91.0–100) % vs. 86.7 (71.6–99.0) %, *P* = 0.052] were similar. Similarly, between the PPOS and the GnRH-ant groups, the fertilisation rate [87.5 (56.3–100) % vs. 60 (50.4–68.9) %, *P* = 0.374], cleavage developmental rate [100 (89.3–100) % vs. 71.4 (58.3–100) %, *P* = 0.237], blastocyst developmental rate [100 (87.5–100) % vs. 71.4 (58.3–100) %, *P* = 0.694], and the number of frozen embryos [5 (2–6) vs. 4 (3–4), *P* = 0.768] were similar (Table 3).

### Factors correlated with ART outcomes

We identified factors correlated with ART outcomes. Factors that potentially correlate with the retrieved number of oocytes were examined. The number of oocytes retrieved (43 patients, 67 cycles) correlated significantly with AMH serum levels (*R*^*2*^ = 0.623, *P* < 0.001) (Supplementary Fig. 1) but did not correlate with age (*R*^*2*^ = 0.004, *P* = 0.619) (Supplementary Fig. 2). The total number of oocytes retrieved, age, and AMH, which may be associated with ART outcomes, were tested for their correlations with fertilisation and embryo development rates. The total number of retrieved oocytes correlated negatively with fertilisation rates (*R*^*2*^ = 0.440, *P* = 0.001) but not with embryonic development rates (*R*^*2*^ = 0.038, *P* = 0.396) (Supplementary Fig. 3). Age showed no correlation with fertilisation rates (*R*^*2*^ = 0.073, *P* = 0.238) or embryonic developmental rates (*R*^*2*^ = 0.015, *P* = 0.593). The serum levels of AMH correlated with fertilisation rates (*R*^*2*^ = 0.305, *P* = 0.009) but not with embryonic development rates (*R*^*2*^ = 0.085, *P* = 0.201) (Supplementary Figs. 1 and 2).

## Discussion

We investigated the efficacy of FP using cryopreserved oocytes and embryos under different COS protocols in patients with cancer or autoimmune diseases before and after gonadotoxic therapy. Our results showed that mature oocytes could be obtained after gonadotoxic treatment, although in smaller numbers, with a comparable oocyte maturation rate. Equivalent ART outcomes were obtained regardless of the COS protocol applied. The number of mature oocytes was correlated with AMH but not with age.

The current recommendations based on the European Society of Human Reproduction and Embryology guidelines state that “For women with reduced ovarian reserve (Bologna criteria, AMH < 0.5 ng/mL), advise needs to be individualised and the value of FP is unclear” [10]. We found that the AMH level and AFC were significantly lower in the post-gonadotoxic treatment group than in the pre-gonadotoxic treatment group. The number of oocytes retrieved in the post-gonadotoxic treatment group was overly low, reflecting its lower ovarian reserve than in the pre-gonadotoxic treatment group. Only 3 (0–4) unfertilised oocytes of fine quality could be retrieved per cycle, and the maturation rate per total number of aspirated oocytes was comparable between the pre- and post-gonadotoxic treatment groups. Additionally, patients in the post-gonadotoxic treatment group underwent 3 (1–4) oocyte retrieval cycles and cumulatively froze 7 (3–7) MII oocytes. FP may be worthwhile after remission, considering there is time to repeat oocyte retrieval after remission, allowing for a sufficient number of eggs to be frozen.

There are a few reports of FP after gonadotoxic treatment. Dolmans et al. (2005) treated 11 patients before or immediately after chemotherapy for cancer using the short protocol and were unable to obtain oocytes from three patients who had received 2–3 cycles of chemotherapy for acute leukaemia [18]. This was likely as oocyte harvest was attempted in these patients within a short time of 4–14 weeks after the first chemotherapy treatment. In 2013, A multicentred French study on post-gonadotoxic treatment cases based on a retrospective questionnaire [11], showed that 28% (14 cases) of patients who underwent emergency in vitro fertilisation had received prior chemotherapy, and only one cycle was cancelled because of a lack of response to COS. However, detailed information on the regimen of gonadotoxic treatment and COS is not provided. Akino et al. performed FP on five patients with haematological cancer treated with a low-risk gonadotoxic regimen and the mean number of eggs retrieved was reported as 3.2 [19]. In contrast, the treatment of our patients included cisplatin and bone marrow transplantation, both of which are highly gonadotoxic, followed by FP after a long interval (Supplementary Table 1). Our results demonstrate that FP is effective even in women with reduced ovarian reserves after gonadotoxic treatment.

In addition, the FP group appeared to be fertile; therefore, a high pregnancy rate may be achieved even if a low number of oocytes are retrieved. Five patients underwent embryo transfer post-remission using frozen embryos before gonadotoxic therapy, resulting in a clinical pregnancy rate of 55.6% (5/9) per single embryo transfer. All five patients achieved clinical pregnancy, and three of the five patients had live births. These results suggest that three oocytes (two embryos) are required to reach 60% cumulative live births in the FP group, which is even smaller than that in infertile patients in their 30 s who require approximately 15 oocytes to reach 60% cumulative live births [20].

The chances of spontaneous conception after remission are reported to be generally limited to patients in their 20 s following hematopoietic stem cell transplantation during childhood [21, 22], suggesting that these patients suffer from POI and tend to lose the chance for pregnancy during their 30 s. Therefore, FP may have some value in expanding family-building options for those who have already undergone gonadotoxic treatments.

It remains unknown whether the drugs used in chemotherapy directly affect germ cells, but there are concerns regarding harvesting of oocytes exposed to chemotherapy and radiation therapy. Data from animal studies indicate that chemotherapy and radiotherapy are mutagenic to germ cells at various stages of gonad maturation [23–27]. On the contrary, gonadotoxic treatments that influence the cell cycle are unlikely to act on an oocyte that is quiescent before entering meiosis. A questionnaire-based survey of cancer survivors treated with cancer treatment prior to conception reports no increased risk of obvious congenital abnormalities in their offspring over an average observation period of approximately 11 years [28]. A few drugs, such as tamoxifen, are teratogenic when administered during pregnancy [29]. Therefore, it is recommended to wait 2 months after the termination of tamoxifen administration before becoming pregnant to allow tamoxifen and its metabolites to be eliminated from the body. The time to safe conception after treatment completion is not precisely known, and the timing of pregnancy approval must be determined individually while considering the half-life and mechanism of each drug.

Gonadotoxic treatment may have an effect even after conception. Pregnancy within one year of gonadotoxic treatment is related to an increased risk of obstetric abnormalities such as preterm delivery and low birth weight [30]. Based on these reports, we attempted embryo transfer more than one year after the last treatment. Among the five patients who underwent embryo transfer, two had miscarriages (40.0%), one had a preterm delivery (20.0%), and two had full-term deliveries (40.0%); these rates are comparable to those in infertile patients who conceived using ART (miscarriage rate 25.9%) [31] and those with a preterm delivery rate between 10–14% [32]. Therefore, both FP and spontaneous conception post-gonadotoxic treatment are safe at approximately one year after treatment. However, the gonadotoxicity of newly developed drugs has not been determined, and there is also a risk of POI if the waiting period is too long. In addition, because egg donation is not permitted in Japan, patients who wish to conceive must use their eggs. Thus, during patient counselling, we provide decision support, being careful not to miss the timing of pregnancy due to POI, noting that there is insufficient evidence to provide reassurance regarding pregnancy.

There are a variety of protocol options for COS. Evidence on the usefulness and safety of using PPOS in FP is limited. Huang et al. (2022) compared 30 cycles of the PPOS protocol with 56 cycles of the GnRH-ant protocol in the total of 86 cycles of FP performed in women with breast cancer and haematological diseases [14]. The results of the study showed that both LH suppression regimens result in a comparable number of matured oocytes with no cancellation of oocyte retrieval, which is consistent with our results. Further studies are needed to confirm the usefulness of PPOS in FP.

An association between AMH levels and the oocyte retrieval number has been reported [33]. In addition, AMH levels are reduced by gonadotoxic treatment [34]. These reports are consistent with our finding that the oocyte retrieval number in the post-gonadotoxic treatment group with relatively low AMH was lower than that in the pre-gonadotoxic treatment group.

In the general infertile population, pregnancy rates are known to decrease with increasing age. In contrast, in our study, no correlation was observed between pregnancy rates and age. A possible reason for this difference is that the patients who underwent FP are not infertile, and thus the effect of age on fertility is likely small. Since age alone is not a sufficient predictor of pregnancy outcome in FP, new observational measures are needed. Detailed analysis of embryo development using a time-lapse system incubator has shown that embryos derived from women of reproductive ages (798 oocytes, ages ≥ 41 years, 191 cycles) grow more slowly than those from younger women (1223 oocytes, age < 30 years, 173 cycles). Specifically, the time for a zygote to form a full blastocyst in embryos derived from younger women (116.79 ± 8.84 h) is shorter than that for embryos derived from older women (126.58 ± 14.25 h) (*P* < 0.001) [35]. Similarly, more detailed analysis using a time-lapse system, such as differences in the interval of the development of the embryos from zygotes to blastocysts, may be able to predict FP pregnancy outcomes and evaluate the efficacy of FP.

In patients at a high risk of gonadal toxicity [36], AMH levels decrease during and after 6 months of chemotherapy. In patients with a low to intermediate risk of gonadal toxicity [36], although AMH levels decrease during treatment, AMH levels recover to pre-treatment levels by six months after chemotherapy completion [34]. Although the degree of reproductive toxicity is evaluated based on the presence or absence of menstrual recovery, it appears that the risk of gonadotoxicity of individual drugs should be assessed by AMH measured before gonadotoxic treatment and six months after completion of chemotherapy. As sufficient oocyte numbers were obtained through repeated oocyte retrieval even after gonadotoxic treatment, aggressive FP is recommended before POI is reached, even if AMH levels are low.

FP was effective in both the pre- and post-gonadotoxic treatment groups, and PPOS was effective in FP for ovarian stimulation. The main limitation of the current study is the relatively small sample size used in the outcome analysis of ART within a single-centre study. There were many frozen oocytes but fewer cases of fertilisation. Both the number of subjects in the post-gonadotoxic treatment group and the lack of investigation of the post-fertilisation outcome of the oocytes obtained could be considered limitations. Our results should be interpreted with caution, as different malignancy types, autoimmune diseases, and therapies were included in the study groups, although their distributions were similar between the groups. Another limitation is that the pregnancy rate per embryo transfer has not been produced in the post-gonadotoxic treatment group. The observation period was short, and many cases did not show remission or result in embryo transfer. Multicentre studies and national registries are needed to assess the likelihood of pregnancy relatively more accurately after the freezing of oocytes and embryos.

## Conclusion

The efficacy and safety of FP in adolescent and young adult patients with cancer and autoimmune diseases were reviewed. Although FP is recommended before gonadotoxic treatment, it must not be excluded when a patient has missed FP during the pre-treatment period. In the post-gonadotoxic treatment group, mature oocytes were obtained via COS and oocyte retrieval, suggesting that oocyte freezing remains effective for FP after gonadotoxic treatment. Thus, FP was effective in both the pre- and post-gonadotoxic treatment groups, and PPOS was effective in FP for COS. However, the levels of AMH and number of oocytes retrieved were significantly lower in the post-gonadotoxic treatment group than in the pre-gonadotoxic treatment group, even though the post-gonadotoxic treatment group consisted of a younger population. Therefore, multiple oocyte retrievals may be required to obtain enough oocytes to achieve a live birth. Increased cooperation with other departments is necessary to verify the efficacy and safety of FP.

### Supplementary Information

**Additional file 1: Supplementary Fig. 1.** Serum levels of anti-Müllerian hormone (AMH) correlates with the number of oocytes retrieved. **Supplementary Fig. 2.** Patient age is not correlated with assisted reproductive technology (ART) outcomes. **Supplementary Fig. 3.** Total oocyte retrieval number is not correlated with assisted reproductive technology (ART) outcomes.

**Additional file 2: Supplementary Table 1.** Participants' characteristics and ART outcome. **Supplementary Table 2.** Clinical outcomes: Characteristics of patients before gonadotoxic treatment. **Supplementary Table 3.** Effects of age, AMH, the total dosage of gonadotropin use, and gonadotoxic treatments on the number of oocytes retrieved. **Supplementary Table 4.** Effects of age, AMH, the total dosage of gonadotropin use, and gonadotoxic treatments on the number of MII oocytes retrieved.

## Data Availability

The data underlying this article will be shared on reasonable request to the corresponding author.
