# Supplementary figures and images for: Fertility preservation after gonadotoxic treatments for cancer and autoimmune diseases
Source: J Ovarian Res. 2023 Aug 10;16:159. doi: 10.1186/s13048-023-01250-x (PMC10416401; doi:10.1186/s13048-023-01250-x)

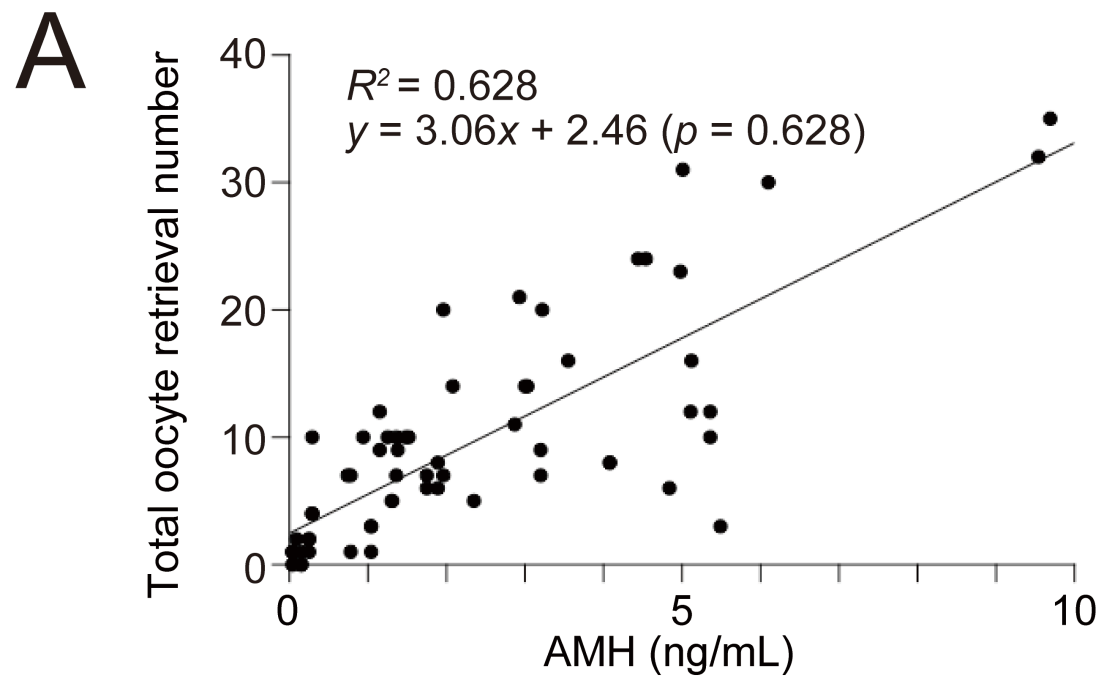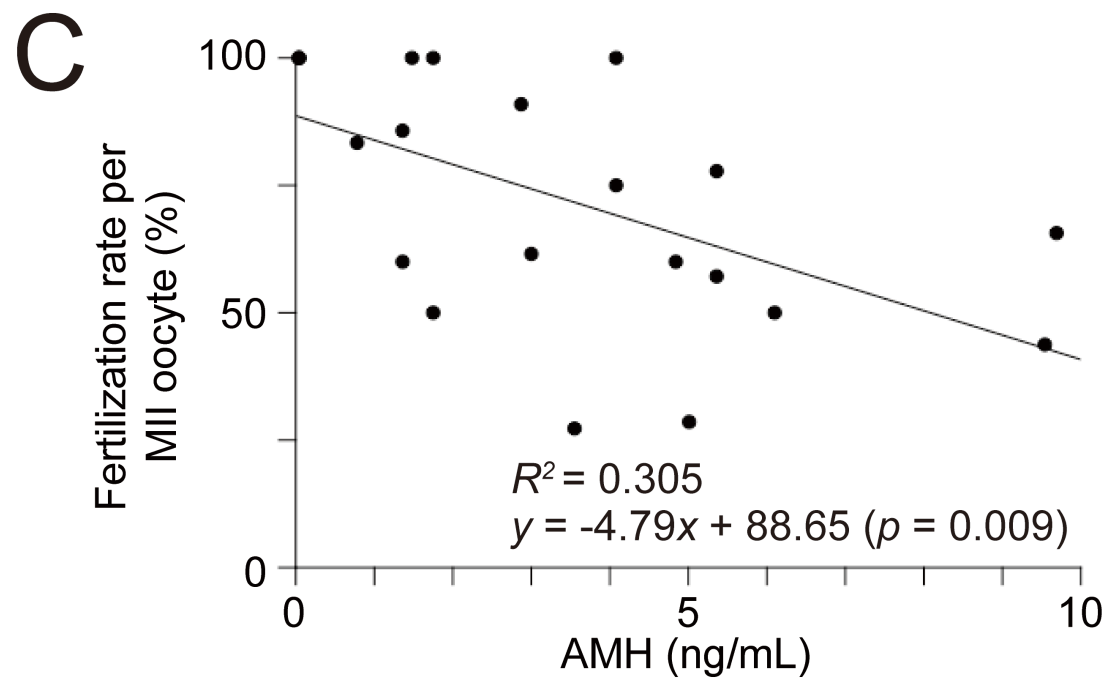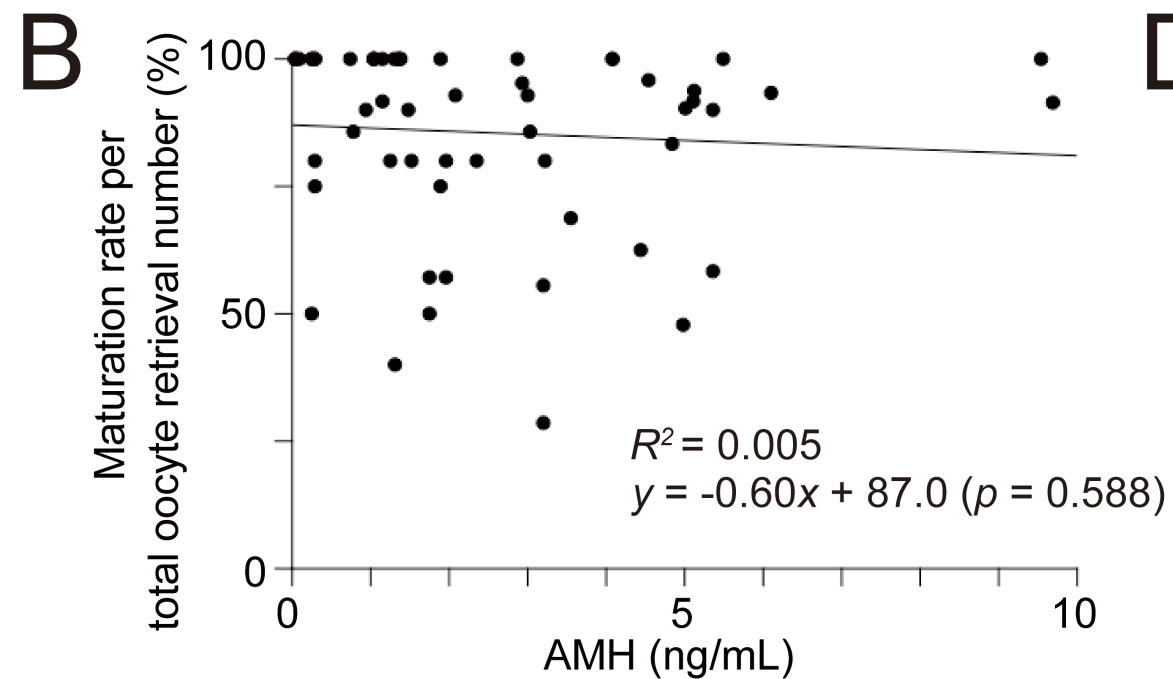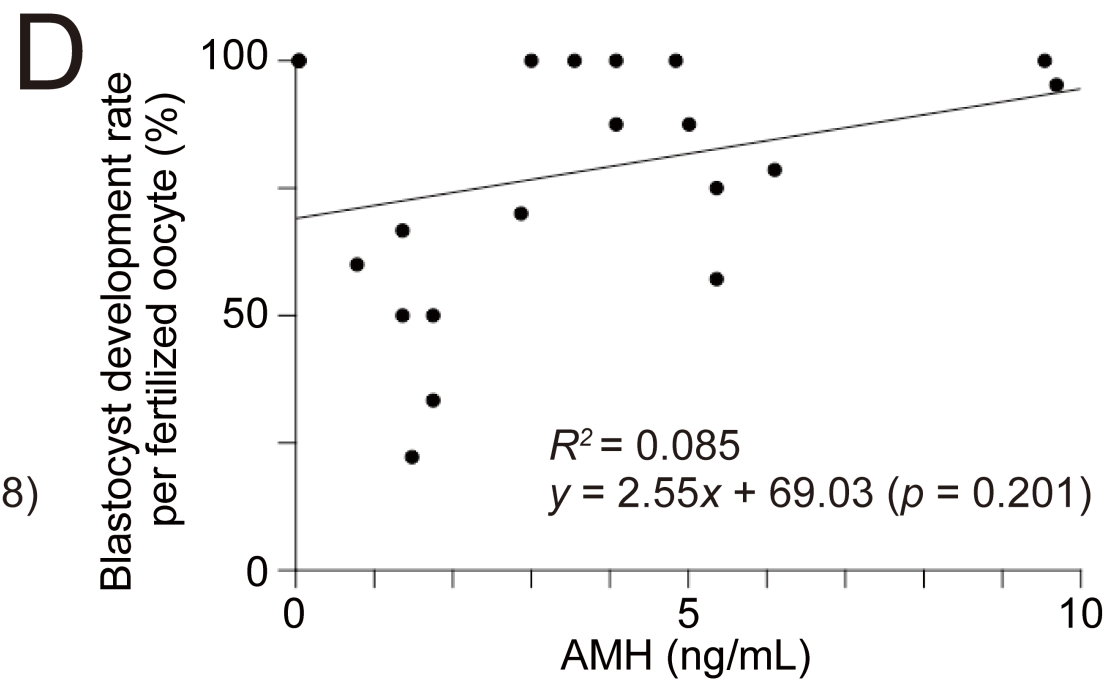

Supplementary Figure 1

**A**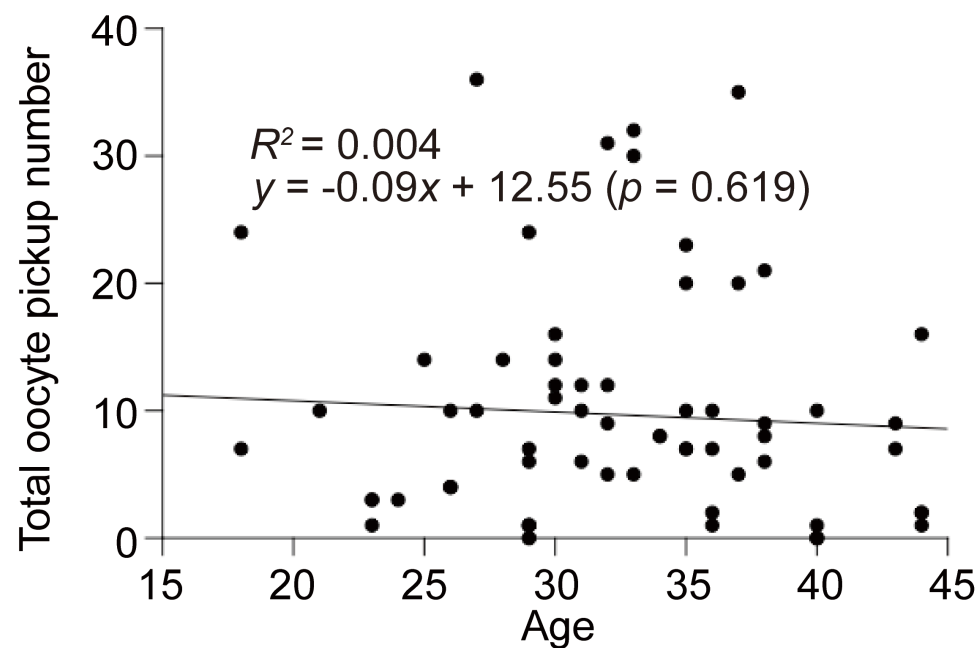**B**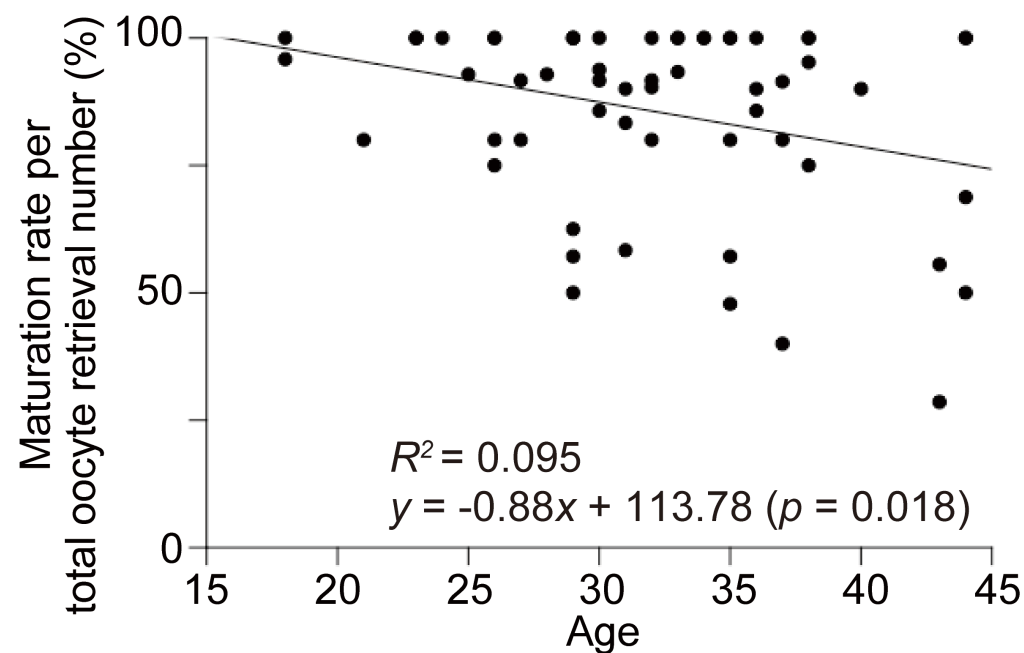**C**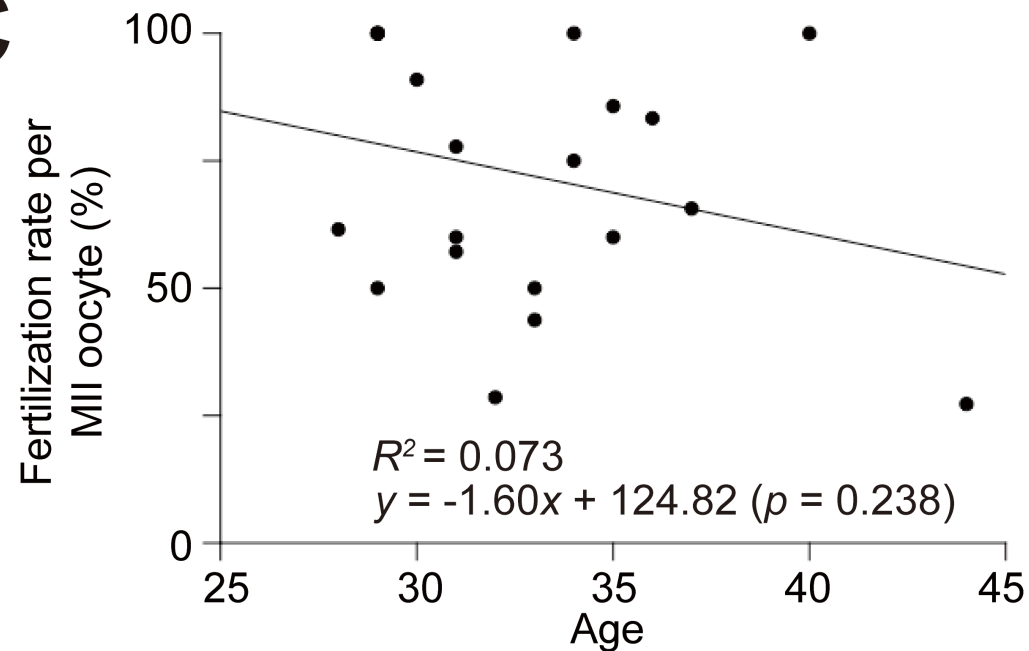**D**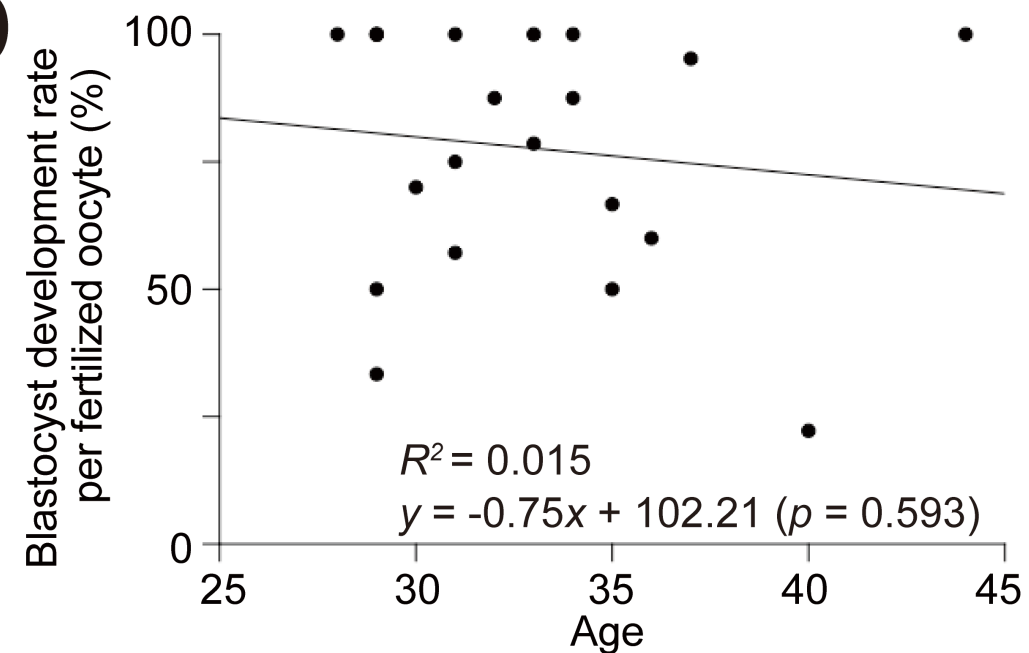

# Supplementary Figure 2

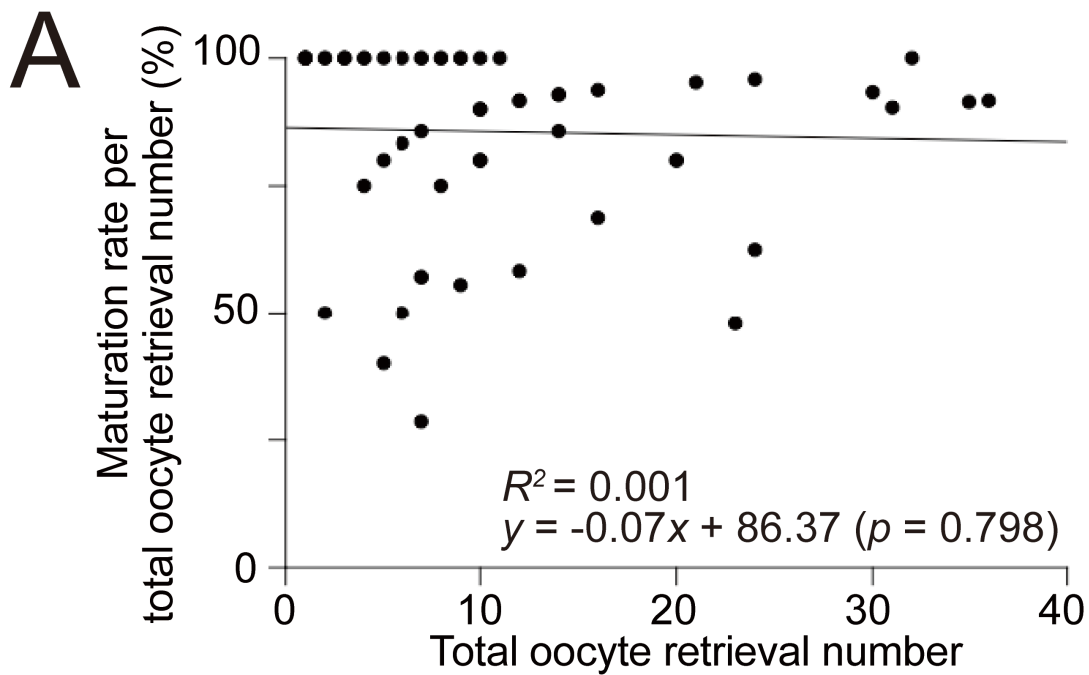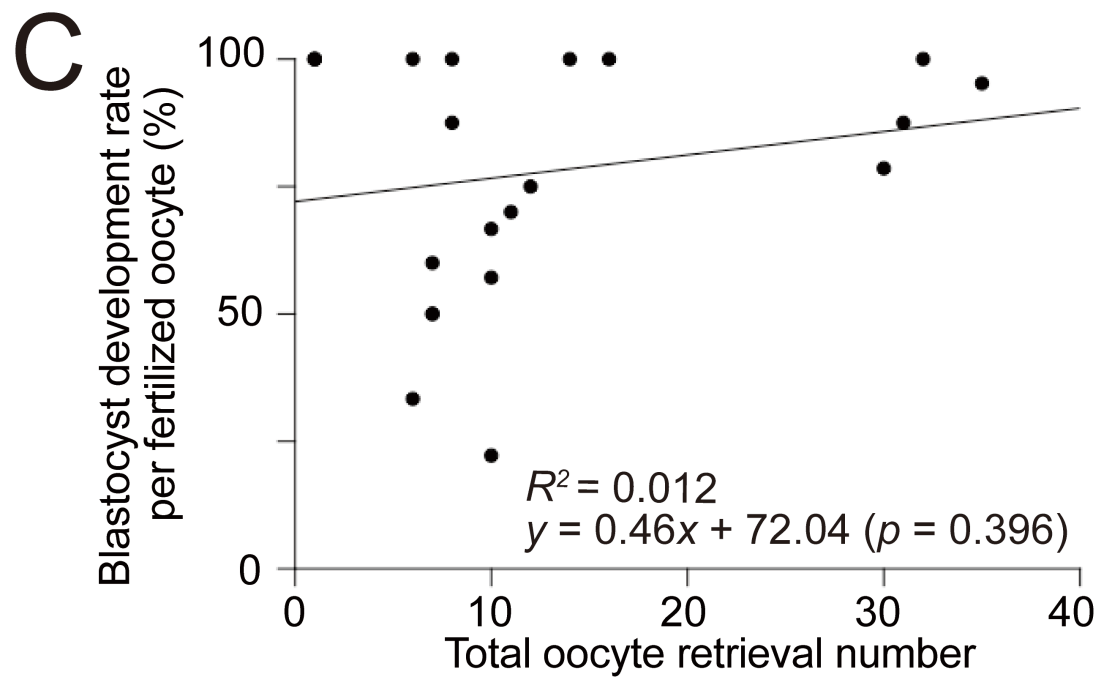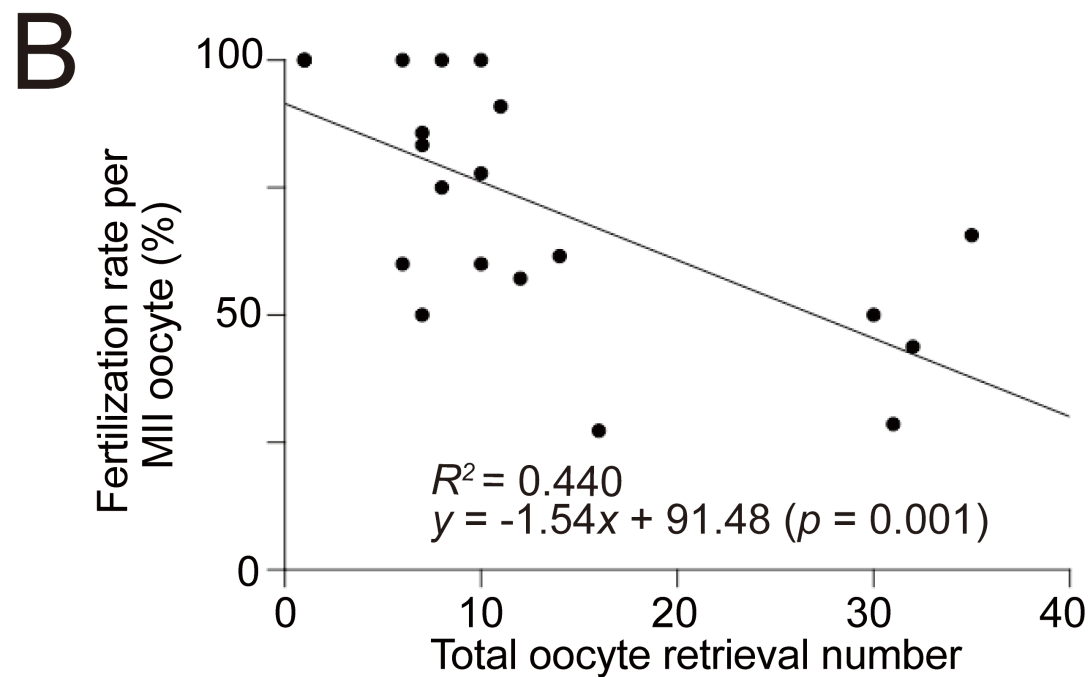

Supplementary Figure 3

Supplement: Supplementary file 1 — Additional file 1: Supplementary Fig. 1. Serum levels of anti-Müllerian hormone (AMH) correlates with the number of oocytes retrieved. Supplementary Fig. 2. Patient age is not correlated with assisted reproductive technology (ART) outcomes. Supplementary Fig. 3. Total oocyte retrieval number is not correlated with assisted reproductive technology (ART) outcomes. [file 13048_2023_1250_MOESM1_ESM.pdf]
